# In depth analysis of genes and pathways of the mammary gland involved in the pathogenesis of bovine *Escherichia coli-*mastitis

**DOI:** 10.1186/1471-2164-12-130

**Published:** 2011-02-28

**Authors:** Bart Buitenhuis, Christine M Røntved, Stefan M Edwards, Klaus L Ingvartsen, Peter Sørensen

**Affiliations:** 1 Department of Genetics and Biotechnology, Faculty of Agricultural Sciences, Aarhus University, Blichers allé 20, P.O. Box 50, DK-8830 Tjele, Denmark; 2 Department of Animal Health and Bioscience, Faculty of Agricultural Sciences, Aarhus University, Blichers allé 20, P.O. Box 50, DK-8830 Tjele, Denmark

## Abstract

**Background:**

Bovine mastitis is one of the most costly and prevalent diseases affecting dairy cows worldwide. In order to develop new strategies to prevent *Escherichia coli*-induced mastitis, a detailed understanding of the molecular mechanisms underlying the host immune response to an *E. coli *infection is necessary. To this end, we performed a global gene-expression analysis of mammary gland tissue collected from dairy cows that had been exposed to a controlled *E. coli *infection. Biopsy samples of healthy and infected utter tissue were collected at T = 24 h post-infection (p.i.) and at T = 192 h p.i. to represent the acute phase response (APR) and chronic stage, respectively. Differentially expressed (DE) genes for each stage were analyzed and the DE genes detected at T = 24 h were also compared to data collected from two previous *E. coli *mastitis studies that were carried out on *post mortem *tissue.

**Results:**

Nine-hundred-eighty-two transcripts were found to be differentially expressed in infected tissue at T = 24 (*P *< 0.05). Up-regulated transcripts (699) were largely associated with immune response functions, while the down-regulated transcripts (229) were principally involved in fat metabolism. At T = 192 h, all of the up-regulated transcripts were associated with tissue healing processes. Comparison of T = 24 h DE genes detected in the three *E. coli *mastitis studies revealed 248 were common and mainly involved immune response functions. KEGG pathway analysis indicated that these genes were involved in 12 pathways related to the pro-inflammatory response and APR, but also identified significant representation of two unexpected pathways: natural killer cell-mediated cytotoxicity pathway (KEGG04650) and the Rig-I-like receptor signalling pathway (KEGG04622).

**Conclusions:**

In *E. coli*-induced mastitis, infected mammary gland tissue was found to significantly up-regulate expression of genes related to the immune response and down-regulate genes related to fat metabolism. Up to 25% of the DE immune response genes common to the three *E. coli *mastitis studies at T = 24 h were independent of *E. coli *strain and dose, cow lactation stage and number, tissue collection method and gene analysis method used. Hence, these DE genes likely represent important mediators of the local APR against *E. coli *in the mammary gland.

## Background

Inflammation of the udder tissue, known as mastitis, has become one of the most costly and prevalent diseases affecting dairy cows worldwide [1]. Mastitis onset has been associated with many different bacterial infections, but the most common are staphylococci, streptococci and coliform bacteria [2]. *Escherichia coli *(*E. coli*) is a prevalent environmental pathogen that routinely colonizes dairy cattle, and represents a significant risk of mastitis [2]. *E. coli*-induced mastitis is characterized as a relatively short-term disease process [3] and induces a distinct acute phase response (APR). The precise response kinetics, however, depend on various biological and environmental factors, such as host diet, *E. coli *strain and dose, and time of infection [4].

The innate immune response is triggered at the earliest stages of infection, as its function is to recognize pathogens that have not been encountered before [5]. Therefore, the innate immune system is effectively the first line of defence against intramammary *E. coli *infection. Many of the cytokines and other inflammatory mediators involved in the innate immune response are characterized by a specific pathogen-dependent expression profile [6]. Gram-negative bacteria, such as *E. coli*, are characterized by an early and high expression of cytokines and other inflammatory mediators in the milk, while Gram-positive bacteria, such as *Staphylococcus aureus *(*S. aureus*), show a lower and/or delayed expression of cytokines and other inflammatory mediators in the milk [6]. Moreover, the inflammatory responsiveness to *E. coli *endotoxin is highly influenced by the cows' lactation stage [7,8]. Recently, the Toll-Like Receptor (TLR)-4 transduction pathway was suggested as a crucial contributor to robust mammary gland immune defence against *E. coli *mastitis [9,10]. This pathway involves induction of cellular inflammatory and apoptotic responses, and eventually leads to the activation of NF-κB factors in resident macrophages, monocytes and epithelial cells [9,10]. Diapedesis of polymorphonuclear neutrophil leukocytes (PMN) is impaired in early lactation, and a reduced neutrophil influx into the mammary gland during the APR is believed to promote the incidence of severe *E. coli *mastitis during this period [11]. Hence, both macrophages and neutrophils are considered as important players in the local APR during *E. coli *mastitis.

To gain a detailed understanding of the defence mechanisms employed by the mammary gland, the genes and gene pathways that are altered in response to the presence of pathogenic bacteria should be identified and characterized. The development of microarray technology has enabled comprehensive screening of defined gene expression profiles in specific tissues, such as the mammary gland. Recently, this type of analysis was carried out on udder tissue collected *post mortem *in dairy cows experimentally infected with medium to high doses of *S. aureus *[12], *S. uberis *[13] and *E. coli *[14,15] intramammary.

The principal focus of our study was on the gene-expressions in udder tissue biopsies collected *ante mortem *from dairy cows during the APR (24 h p.i) and the chronic stage (192 h p.i) of the *E. coli *infection when using a low inoculation dose of *E. coli *in early lactation. The aim being to identify the global mammary gland gene expressions and gene pathways associated with bovine *E. coli *mastitis during the acute and chronic stage of the infection in early lactating dairy cows. Furthermore, the aim was to identify common genes involved in the local mammary gland APR between our study and two other gene-expression studies on *E. coli *mastitis [14,15] independent of *E. coli *strain and dose, cow's lactation stage and number, tissue collection method and gene analysis used in the studies.

## Results

### Clinical examinations and para-clinical measurements

All challenged cows (n = 16) developed *E. coli *mastitis and were free of infections with other major udder pathogens during sampling. *E. coli *was identified in the infected quarters of all 16 cows at 24 h after inoculation. At T = 120 h p.i., *E. coli *was present in variable concentrations in eight of the cows, and by 192 h all cows had cleared the infection.

**Figure 1:**
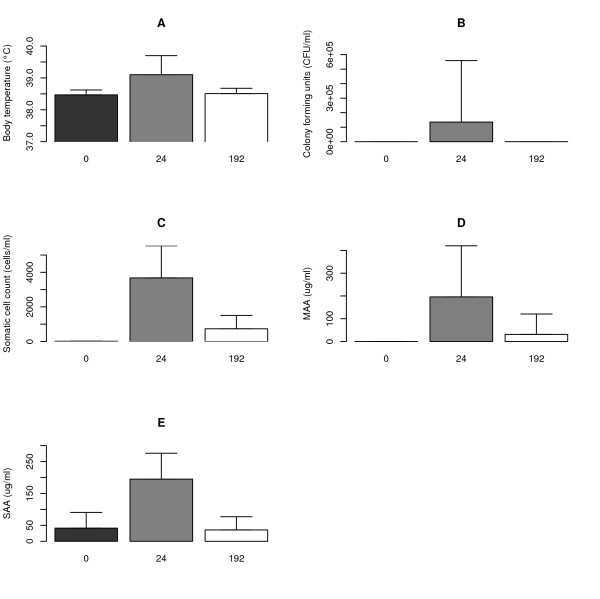
**Overview of the clinical examination and para-clinical measurement of the cows infected with *E. coli *at T = 0 h (black), T = 24 h (grey) and T = 192 h (white)**. The bars correspond to the mean values of the trait, while the whiskers on the bars correspond to the standard deviation. A: body temperature of the cows (°C), B: number of *E. coli *bacteria in the milk (CFU/ml), C: somatic cell count in the milk (cells/ml), D: concentration of Milk Amyloid A in milk (μg/ml), and E: concentration of Serum Amyloid A in blood (μg/ml).

Body temperature, colony forming units (CFU/ml) of *E. coli *in the milk, milk somatic cell count (SCC), and the concentrations of Milk Amyloid A (MAA) and of Serum Amyloid A (SAA) in blood in response to *E. coli *infection at time points T = 0 h, T = 24 h, and T = 192 h are presented in Figure 1. In the acute phase (T = 24 h), the body temperature, *E. coli *CFU in the milk, SCC, concentrations of MAA in milk and SAA in blood were significantly increased, as compared to the data from T = 0 h and T = 192 h. These findings were consistent with symptomology of *E. coli*-induced mastitis, and confirmed the model system. At T = 192 h, even though all cows had successfully cleared the *E. coli*, SCC and MAA concentrations remained higher than at T = 0 but to a lesser extent than observed at T = 24 h. These findings confirmed the establishment of the chronic stage.

### Acute phase response

In total, 982 DE transcripts were detected (*P *< 0.05) at this stage, of which 684 corresponded to 609 annotated genes [Additional file 1: Supplemental Table S1]. Hierarchical clustering of the DE transcripts revealed two main clusters (up-regulated and down-regulated).

### Up-regulated genes

Cluster 1 (up-regulated) contained 699 of the DE transcripts, representing 551 distinct genes. Hypergeometric gene set analysis based on the GO information revealed overrepresentation of 92 gene sets [Additional file 2: Supplemental Table S2]. Of these gene sets, 43 were directly related to immune response. GO identifiers were related to inflammatory response (GO:0006954), chemotaxis (GO:0006935), immune response (GO:0006955), leukocyte migration (GO:0050900), response to lipopolysaccharide (GO:0032496), and TLR signalling pathway (GO:0002224). In contrast, the hypergeometric gene set analysis based on the KEGG information revealed 18 gene sets (Table 1). These were related to chemokine signalling pathway (KEGG04062), TLR signalling pathway (KEGG04620), leukocyte transendothelial migration (KEGG04670), cytokine-cytokine receptor interaction (KEGG04060), natural killer (NK) cell-mediated cytotoxicity (KEGG04650), and RIG-I-like signalling pathway (KEGG04622). Among the DE transcripts in this cluster the genes encoding chemokine (C-C motif) ligands (CCL3, CCL4, CCL5, CCL16, CCL19), chemokine (C-X-C motif) ligands (CXCL2, CXCL5, CXCL16), interleukin 1β (IL1B), interleukin 8 (IL-8), interleukin receptors (IL1R1, IL2RA, IL2RG, IL4R, IL6R, IL8R), interferon regulatory factor 9 (IRF9), neutrophil cytosolic factors (NCF1, NCF2), and Toll-like receptors (TLR2, TLR4) were represented.

**Table 1:** Significant KEGG identifiers detected based on the differentially expressed transcripts in cluster 1 for the acute phase response to *E. coli *infection*

KEGG ID	*P *value	Odds Ratio	Exp Count	Count	Size	Term
4062	2.98E-09	5.571349	5.027606	22	106	Chemokine signaling pathway
4620	4.87E-06	6.066348	2.513803	12	53	Toll-like receptor signaling pathway
4670	2.71E-05	4.568835	3.414978	13	72	Leukocyte transendothelial migration
4060	9.62E-05	3.722762	4.363583	14	92	Cytokine-cytokine receptor interaction
4650	0.000254	4.473538	2.656094	10	56	Natural killer cell mediated cytotoxicity
4514	0.000518	3.714061	3.414978	11	72	Cell adhesion molecules (CAMs)
4622	0.000743	5.504747	1.565198	7	33	RIG-I-like receptor signaling pathway
4666	0.000873	4.103185	2.561233	9	54	Fc gamma R-mediated phagocytosis
4662	0.001521	4.767827	1.754919	7	37	B cell receptor signaling pathway
4612	0.002095	4.468453	1.84978	7	39	Antigen processing and presentation
4920	0.002438	4.332374	1.89721	7	40	Adipocytokine signaling pathway
4210	0.002843	3.718903	2.466373	8	52	Apoptosis
4610	0.00426	3.861615	2.086931	7	44	Complement and coagulation cascades
3050	0.00727	3.941793	1.754919	6	37	Proteasome
4630	0.00791	2.831847	3.509838	9	74	Jak-STAT signaling pathway
5340	0.008959	5.797582	0.853744	4	18	Primary immunodeficiency
564	0.046285	3.241129	1.375477	4	29	Glycerophospholipid metabolism
5200	0.04726	1.759624	7.778561	13	164	Pathways in cancer

*A hypergeometric gene set enrichment test was performed based on cluster 1 of the contrast T24 *vs*. C24. Overrepresentation of gene sets defined by the KEGG database was tested using the Fisher's exact test. A gene set was considered significant if *P *< 0.05.

### Down-regulated genes

Cluster 2 contained 229 DE transcripts, representing 125 known genes that were down-regulated in response to *E. coli *infection. The hypergeometric gene set analysis based on the GO information revealed over representation of 44 gene sets mainly related to fat metabolism [Additional file 3: Supplemental Table S3], including fatty acid biosynthetic process (GO:0006633), lipid metabolic process (GO:0006629), and fatty acid metabolic process (GO:00031). The hypergeometric gene set analysis based on the KEGG information revealed over representation of 7 gene sets, of which three were related to fatty acid synthesis: fatty acid metabolism (KEGG00071), fatty acid biosynthesis (KEGG00061), and glycerolipid metabolism (KEGG00561) (Table 2). Among the DE transcripts in this cluster, genes encoding dehydrogenase/reductase (SDR family) member 3 (DHRS3), fatty acid synthase (FASN), fat storage-inducing transmembrane protein 2 (FITM2), lipase maturation factor 1 (LMF1), and lipoprotein lipase (LPL) were represented.

**Table 2:** Significant KEGG identifiers detected based on the differentially expressed transcripts in cluster 2 for the acute phase response to *E. coli *infection*

KEGG ID	*P *value	Odds Ratio	Exp Count	Count	Size	Term
830	0.00011	19.46957	0.257269	4	24	Retinol metabolism
71	0.002574	12.51056	0.278708	3	26	Fatty acid metabolism
1100	0.016481	2.126324	6.817621	13	636	Metabolic pathways
61	0.021326	93.55556	0.021439	1	2	Fatty acid biosynthesis
980	0.029004	8.222903	0.267988	2	25	Metabolism of xenobiotics by cytochrome P450
561	0.031206	7.879108	0.278708	2	26	Glycerolipid metabolism
982	0.035802	7.270856	0.300147	2	28	Drug metabolism - cytochrome P450

*A hypergeometric gene set enrichment test was performed based on cluster 2 of the contrast T24 *vs*. C24. Overrepresentation of gene sets defined by the KEGG database was tested using the Fisher's exact test. A gene set was considered significant if *P *< 0.05.

### Chronic stage response

In total, only two DE transcripts (Bt.12553.1.S1_at and Bt.7165.1.S1_at) were detected (*P *< 0.05). These encode haptoglobin (HP) and chemokine (C-X-C motif) ligand 5 (CXCL5), respectively.

### Overlap of DE genes between live tissue and related analysis in necrotic tissue

The DE genes were compared to two other related studies on gene expression in response to *E. coli *infection in the udder [14,15]. In [Additional file 4: Supplemental Figure S1], the overlap of statistically significant DE genes at T = 24 h p.i. is presented. A group of 248 genes was represented in all three studies [Additional file 5: Supplemental Table S4]. Among this group are immune-related genes, namely those encoding chemokine (C-C motif) ligands (CCL4, CCL19), chemokine (C-X-C motif) ligands (CXCL2, CXCL5, CXCL16), intercellular adhesion molecules (ICAM1, ICAM3), immediate early response (IER3, IER5), interferon-induced transmembrane proteins (IFITM1, IFITM3), interferon regulatory factor 9 (IRF9), interleukins (IL1B, IL8), interleukin receptors (IL2RG, IL8RB, IL10RB), haptoglobin (HP), neutrophil cytosolic factors (NCF1, NCF2, NCF4), S100 calcium binding proteins (S100A2, S100A8, S100A9, S100A12), serum amyloid A 3 (SAA3), Toll-like receptors (TLR2, TLR4), and tumour necrosis factor receptor superfamily members (TNFRSF1A, TNFRSF6B). The hypergeometric gene set analysis based on the KEGG information revealed overrepresentation of 15 gene sets, of which one was related to fatty acid metabolism (glycerolipid metabolism (KEGG00561)) while the 14 other KEGG identifiers were related to the immune response: chemokine signalling pathway (KEGG04062), leukocyte transendothelial migration (KEGG04670), natural killer cell-mediated cytotoxicity (KEGG04650), Toll-like receptor signalling pathway (KEGG04620), and RIG-I-like receptor signalling pathway (KEGG04622) (Table 3).

**Table 3:** Significant KEGG identifiers detected based on the differentially expressed genes between three studies of *E. coli *infection in the bovine udder*

KEGG ID	*P *value	Odds Ratio	Exp Count	Count	Size	Term
4062	6.05E-08	6.785196	2.70837	15	106	Chemokine signaling pathway
4670	1.73E-06	7.273962	1.839648	11	72	Leukocyte transendothelial migration
4650	1.02E-05	7.646809	1.430837	9	56	Natural killer cell mediated cytotoxicity
4620	5.14E-05	7.058635	1.354185	8	53	Toll-like receptor signaling pathway
4060	0.00053	4.306462	2.350661	9	92	Cytokine-cytokine receptor interaction
4622	0.001365	6.982249	0.843172	5	33	RIG-I-like receptor signaling pathway
4514	0.002293	4.237402	1.839648	7	72	Cell adhesion molecules (CAMs)
4662	0.00231	6.105769	0.945374	5	37	B cell receptor signaling pathway
4920	0.003278	5.579882	1.022026	5	40	Adipocytokine signaling pathway
4210	0.01013	4.147677	1.328634	5	52	Apoptosis
4666	0.011834	3.977177	1.379736	5	54	Fc gamma R-mediated phagocytosis
4610	0.025259	3.88	1.124229	4	44	Complement and coagulation cascades
561	0.027695	5.044241	0.664317	3	26	Glycerolipid metabolism
4142	0.038281	2.85764	1.865198	5	73	Lysosome
4630	0.040239	2.815796	1.890749	5	74	Jak-STAT signaling pathway

*A hypergeometric gene set enrichment test was performed based on overlapping genes between the study described in this paper, Mitterhuemer *et al*. [14], and Rinaldi *et al*. [15]. Overrepresentation of gene sets defined by the KEGG database was tested using the Fisher's exact test. A gene set was considered significant if *P *< 0.05.

## Discussion

In this study, we analyzed the global gene expression changes in the bovine mammary gland that occurred during the acute phase and chronic stage of experimental *E. coli *mastitis in dairy cows in early lactation. Tissue samples were collected with a biopsy pistol, which made it possible to sample the same *E. coli*-infected cows *in vivo *at two different time periods.

The acute phase was verified by the presence of high *E. coli *CFU/ml in milk, fever, increased SCC and the presence of acute phase proteins MAA and SAA in milk and blood, respectively. Marked changes in gene expression were detected in the acute stage at 24 h p.i. In agreement with results from other related studies the up-regulated genes were related to the immune response, with the majority of genes involved in the induction and regulation of the local inflammatory response and the APR [9,16]. Among the top 10 KEGG pathways associated with these genes were pathways involved in chemokine signalling, Toll-like receptor signalling, leukocyte transendothelial migration, cytokine-cytokine receptor interaction, cell adhesion and Fc gamma R- mediated phagocytosis. This finding indicates the importance of recruitment and activation of macrophages and neutrophils to sites of infection during acute *E. coli *mastitis [17,18].

In the acute phase, the group of down-regulated genes in the mammary gland was mainly associated with the lipid metabolism and fatty acid pathways. It is well known that cows with *E. coli *mastitis experience decreased milk production [19,20]; however, the affected molecular mechanisms related to fat synthesis in those cows has been undefined [21]. The results presented in this paper, in conjunction with the results of Mitterhuemer *et al*. [14], clearly demonstrate that *E. coli *infection of the mammary gland reduces the expression of genes involved in fat metabolism. However, further research is needed to verify whether these genes actually influence the total amount of fat secretion in the milk or instead influence the fat percentage in the milk.

In the chronic, subclinical stage examined at 192 h p.i., all cows were found to have cleared the *E. coli *bacteria and re-established normal body temperatures. Yet, the increased SCC and increased concentrations of MAA in milk were maintained. At this stage, gene expression had returned to control levels, with the notable exception of the genes encoding for the acute phase protein haptoglobin (HP) and the chemokine CXCL5. Both genes were found to be up-regulated at the APR and chronic stage response. CXCL5 expression on the surface of eosinophils facilitates their ability to recruit and activate CXC receptor 2 (CXCR2)-bearing cells, such as neutrophils, to the site of inflammation. In this manner, CXCL5 can influence tissue remodelling [22], such as would be expected in recovering udder tissue. It is likely that more up-regulated genes associated with tissue modelling could have been identified if the samples had been collected earlier. However, as the biopsy procedure itself causes tissue damage and bleeding, there is a limit to how often a biopsy can be collected without influencing the gene expression results.

By comparing our study's results with those from two recent array studies using *post-mortem *tissues from *E. coli *infected cows [14,15], we identified a particularly strong dataset of the statistically significant genes and genetic pathways present in the mammary tissue during the acute phase at 24 h. All three studies were conducted in the Holstein Friesian breed, but differed with regard to *E. coli *strain and dose, cows' lactation number and stage, and the sampling procedure. In our study, a low dose (20-40 CFU/quarter) model was established in primiparous dairy cows in early lactation, and tissue samples were collected *in vivo *using biopsies at time points 24 h and 192 h p.i. in the lobuli-alveoli area. Rinaldi *et al*. [15] used a medium dose (400 CFU/quarter) in a two-step inoculation model in two quarters, with a 12 h time-delay in multiparous cows in late lactation; these tissue samples were collected *post-mortem *from euthanized cows at 12 h and 24 h p.i. from four different portions of the udder. Mitterhuemer *et al*. [14] also used a medium dose model (500 CFU/quarter), but relied on mid-lactation cows and collected tissue samples *post-mortem *at 6 h and 24 h p.i. from a less well-defined area of the udder. Furthermore, the gene analysis platforms used were different between each of the studies. All of the factors mentioned above can influence disease kinetics and gene expression levels in the mammary gland at 24 h p.i. This fact may explain why each study found a unique set of differentially expressed genes, as shown in the non-overlapping areas of the Venn-diagram [Additional file 4: Supplemental Figure S1].

A large group of DE genes were common between the three studies and were determined to be unaffected by *E. coli *strain, dose, cow factors, sampling method and gene expression analysis ([Additional file 4: Supplemental Figure S1], [Additional file 5: Supplemental Table S4]). The gene sets obtained from the analysis of the common 248 DE genes were mainly related to the immune response and are associated with the response to *E. coli *mastitis [14,15] (Table 3). However, in all three studies two of the 15 pathways were of interest because they are principally related to intracellular infections (e.g. viruses, protozoa and bacteria).

The first pathway, the natural killer cell-mediated cytotoxicity pathway (KEGG04650), ranked fifth on our list (Table 1) and third in the comparison between the *E. coli *gene expression studies (Table 3). The NK cells are innate lymphocytes, which function to eliminate all aberrant cells which have completely lost, or express insufficient amounts of, MHC class I molecules; virally-infected cells or tumorigenic cells are examples of such targets [23,24]. Moreover, NK cells are major cytokine producers and participate in the onset and shaping of the adaptive immune response. So far, bovine NK cells have been investigated in relation to intracellular infections with protozoa and mycobacteria [24]. The mechanisms underlying NK cells role(s) in the defence against extracellular bacteria, such as *E. coli*, remains a question of interest. NK cell lyses of damaged apoptotic and necrotic cells is one possibility. Furthermore, NK cells might be involved in a response to *E. coli *via the dendritic cells (DC) [25]. The main role of the DC is processing antigens and presenting antigens to other cells, namely the antigen presenting cells (APC). In this manner, the NK cells may play a role in maintaining homeostasis of the immune response during bacterial infections by regulating the APC amount in the secondary lymphoid organs [25]. In [Additional file 5: Supplemental Table S4], CD69, a DC-induced activation marker of NK, as well as IL2RA and IL2RG, which are part of the IL2 receptor complex on lymphocytes and NK cells, are found in the natural killer cell-mediated cytotoxicity pathway. This suggests a possible function of NK cells in the immune defence process directed against *E. coli *in the mammary gland. To our knowledge, NK and DC have not been identified in healthy or inflamed mammary gland tissues from dairy cows; and, it remains to be shown if and how they are recruited from the blood compartment or local lymph nodes to the mammary gland.

The second interesting pathway, the Rig-I-like receptor signalling pathway (KEGG04622), ranked sixth on our list. Cells of the innate immune system, such as macrophages and DC, express a number of pattern-recognition receptors (PRR) that specifically recognize unique pathogen-associated molecular patterns (PAMP). In this study, as well as in others, TLR2 and TLR4 are characterized as being significantly influenced by *E. coli *mastitis. Comparison of the three related studies also provided evidence for involvement of molecules functioning in the RIG-I-like receptor pathway [Additional file 5: Supplemental Table S4]. The gene-encoded products include receptors such as RIG-I, Mda5, and LGP2, which are cytoplasmatic proteins that recognize viral DNA and RNA [26]. Following recognition of viral-associated molecular patterns, the RIG-I-like receptors stimulate the synthesis of multiple cytokines including the group of interferon type 1 cytokines and pro-inflammatory cytokines that influence protein synthesis, growth arrest and apoptosis. Furthermore, the RIG-I-like receptors enhance DC maturation, NK cell activation, antibody production and differentiation of cytotoxic T-lymphocytes, which in turn facilitate the adaptive immune response [26]. Whether the RIG-I-like receptors are up-regulated directly from contact with *E. coli *components in the mammary gland or indirectly via cross-induction through other TLRs is yet unknown.

The exact role of the natural killer cell-mediated cytotoxicity pathway (KEGG04650) and the Rig-I-like receptor signalling pathway (KEGG04622) in the response to *E. coli *infection of the bovine udder is still unresolved. The fact that identical pathway-associated genes were similarly regulated during the APR in three independent studies indicates that further investigation of these pathways and their related cells will provide significant insights into the pathogenesis of *E. coli *mastitis. Furthermore, this work sets stage for additional work to determine whether patterns of gene expression associated with udder infections with Gram positive bacteria e.g. *S. aureus *and *S. uberis *differs from that of Gram negative infection and whether the timing of gene changes are similar.

## Conclusions

In conclusion, we have identified multiple DE genes involved in the APR and two genes in the chronic stage response to *E. coli*-induced mastitis in the mammary gland of dairy cows in early lactation. The up-regulated genes from the local APR were involved in the immune response, while the down-regulated genes were primarily involved in fat metabolism. Comparison of the DE genes in the APR of our study with those detected in two other related gene expression studies on *E. coli *mastitis revealed a common set of 248 DE genes existed despite differences in *E. coli *strain and dose, cow lactation stage and number, tissue collection method and gene analysis method. Based on the annotation and gene pathway analysis it is suggested that these genes play a central role in general host defence against *E. coli *infections in the mammary gland.

## Methods

### Animals and Treatment

Sixteen, healthy primiparous Danish Holstein-Friesian cows were challenged intramammarily with *E. coli *at four to six weeks after parturition. Prior to disease challenge, the general health and udder health of a total of 24 dairy cows were evaluated based on body temperature, white blood cell count (WBC), glutaraldehyde test, Californian Mastitis Test (CMT; (Kruuse, Marslev, DK)) and bacteriological examinations of foremilk samples. Only those cows with normal body temperature and WBC, negative glutaraldehyde test, low CMT test (range 1-5) and found to be free from major mastitis pathogens were used in the study. Tissue quarters for *E. coli *inoculation and biopsy were selected based on CMT scores (≤2) and SCC in foremilk by using the portable DeLaval Cell Counter (DCC; DeLaval, Tumba, Sweden) (range 1-6000 × 10^3 ^cells/ml). The front quarter with the lowest SCC (< 27000 cells/ml) was used for the *E. coli *inoculations. Control quarters were chosen based upon bacteriological examinations conducted prior to *E. coli *inoculation and on the quarter foremilk SCC at 24 h (<181000 cells/ml) and 192 h (<215000 cells/ml). The day prior to the *E. coli *inoculation, sterile Micro-Renathane polyvinyl catheters were inserted into the jugular vein and flushed with a sterile 0.9% NaCl solution containing 50 IU Na-heparin (Loevens Kemiske Fabrik, Ballerup, Denmark).

The cows were housed in a traditional straw-bedded tie stall barn, where they were individually fed and given free access to water. A total mixed ration (TMR) diet including vitamins and minerals was fed *ad libitum *twice a day in equal portions at 0800 and 1530 (24 hr clock). The cows were milked at 0600 and again at 1700.

All procedures involving animals were approved by the Danish Animal Experiments Inspectorate and complied with the Danish Ministry of Justice Laws concerning animal experimentation and care of experimental animals. Inspection was carried out by members of the Danish Animal Experiment committee during the acute stage of the disease.

### Preparation and inoculation of *E. coli*

The *E. coli *used was a Danish field isolate (k2bh2) isolated from a cow with severe, acute mastitis (kindly donated by Dr. Helle Daugaard Larsen, formerly of the Danish Institute for Food and Veterinary Research, Copenhagen, Denmark). All procedures involving handling of the *E. coli *inoculums were conducted in a laminar air flow (LAF) bench under sterile conditions. Prior to the inoculation, *E. coli *was grown overnight at 37°C in brain heart infusion medium (BHI; Merck, VWR - Bie & Berntsen, Denmark) in a 200 rpm-shaking incubator (Stuart Orbital incubator s150; VWR - Bie & Berntsen). The following day, the bacteria suspension was tested for purity on two non-selective enriched agars: mastitis blood agar plates (BA; Steins Eurofins A/S, Holstebro, Denmark) and tryptic soya agar (TSA; BD Difco™ - BD Denmark A/S) and for *E. coli *on a selective coliform MacConkey agar (MCA; Merck). The *E. coli *suspension was transferred to fresh BHI medium (1:10 dilution) and incubated for and additional 3 h at 37°C. Optimal bacteria growth was tested by measuring the OD (600 nm; value >1.8) on a spectrophotometer (Gene-Quant Pro; Amersham Pharmacia Biotech, Uppsala, Sweden). From then on, the *E. coli *suspension was kept on ice. Centrifugation was performed at 14000 rcf, 4°C for 20 min (Sorvall, RC6+ centrifuge; Axeb Danmark A/S, Albertslund). The bacteria pellet was washed twice and resuspended in 15 ml of cold endotoxin-free 0.9% NaCl (Løven Apotek, Hammershøj, Denmark), and 10-fold dilutions were made from this suspension. The number of washed bacteria in the dilutions was counted using the plate count agar (PCA) method on small TSA and MCA plates. The inoculum was kept overnight at 4°C. The following morning, the number of colony-forming units (CFU)/ml was counted using a manual colony counter. Then, *E. coli *was diluted in sterile 0.9% endotoxin-free NaCl to obtain 2-4 CFU/ml. Each dairy cow was inoculated with 10 ml of 0.9% pyrogen-free NaCl solution containing ~20-40 CFU live *E. coli *in one front quarter immediately after evening milking (T = 0 h). The control quarter was not inoculated. Each teat was disinfected twice with cotton wool pre-wetted with 70% ethanol. The *E. coli*-NaCl solution was infused into the gland with a sterile teat cannula and the quarter was thoroughly massaged. After the inoculation, the remaining bacteria suspension was retested in the laboratory to determine *E. coli *number by using large agar plates with TSA and MCA to accommodate 1 ml volume.

### Clinical examinations, milk and blood sampling, and laboratory analysis

Clinical and para-clinical measurements were recorded throughout the experiment. The body temperature, *E. coli *CFU/ml milk, SCC, Milk Amyloid A (MAA) concentration in the milk and the concentration of Serum Amyloid A (SAA) in the blood measured at 0, 24, and 192 h relative to the time of *E. coli *inoculation are reported here.

#### Bacteriological analysis of milk

Ten ml of foremilk samples were aseptically collected from the *E. coli *infected quarter and negative control quarter prior to infection (0 h) and immediately before the udder biopsies were collected at 24 h and 192 h. Quantification of *E. coli *(CFU/ml) was conducted on MCA plates using serial dilutions (1:10) of the milk samples in 9 ml of 0.9% NaCl. From each dilution, 0.1 ml was spread on an MCA plate and incubated for 24 h at 37°C, after which the number of pink colonies were counted using a manual colony counter. In addition, 10 μl aliquots of foremilk were cultured on BA and TSA for 48 h at 37°C in order to rule out the presence of other mastitis pathogens. The minor mastitis pathogen, coagulase negative *staphylococcus spp*., was found in three control quarters at 24 h after inoculation; specifically, two cows presented with increased SCC and these two quarters were excluded from the array analysis.

#### SCC

Foremilk samples were analyzed for SCC as previously described.

#### MAA and SAA

MAA measurements were carried out as previously described [27]. Blood sampling and SAA measurements were carried out as previously described [28].

### Udder biopsies

Udder biopsies were collected from the infected quarter and a healthy control quarter of each cow at 24 h (acute stage: T24; C24) and 192 h (chronic: T192; C192) after the *E. coli *challenge. Sampling was conducted after the evening milking using the following procedure: The cows were immobilized in their tie stall using a halter and rope. The skin surface of each quarter was thoroughly washed and dried. A mild sedation injection of 0.1 ml Domosedan^® ^Vet per 100 kg body weight (10 mg/ml Detomidin; Orion Pharma, Espoo, Finland) was administered intravenously. A skin area of 10 × 10 cm in the middle of each quarter (lobuli-alveolar gland region) was shaved and disinfected twice with 70% ethanol. Local anesthetic consisted of xylocaine (1% lidocaine) (Astra Zeneca A/S, Albertslund, Denmark) that was sprayed onto the shaved skin area. After 10 min, a 0.3-0.6 cm long incision was made in the udder skin with a scalpel, trying to avoid blood vessels visible under the skin. Five udder biopsies were collected at the incision point using a biopsy pistol developed for obtaining biopsies in humans (Manan Automatic Biopsy System; Marmon/MDTech, Gainesville, FL). The needle of the biopsy pistol was 14 gauge with a 17 mm notch, which collected 10-15 mg tissue per biopsy. Bleeding from the incision wound was allowed briefly, after which pressure with sterile cloth tampons were placed on the incision areas to stop bleeding and avoid hematoma formation. After the udder biopsies had been collected, cows were administered a prophylactic antibiotic treatment against infection with Gram-positive bacteria by intramuscular injection of 30 ml of Penovet^® ^vet (300000 IE benzylpenicillinprocain/ml; Boehringer Ingelheim Danmark A/S, Copenhagen, Denmark). The udder biopsies were snap frozen in liquid nitrogen and transported to the laboratory where the tissue was stored at -80°C until RNA extraction.

### Expression profiling using microarrays

The number of samples used in the microarray analysis was: T24, 12; C24, 9; T192, 14; and C192, 14.

Isolation and labelling of RNA and microarray processing was performed as described previously [29]. RNA from the udder biopsies was isolated using Trizol Reagent (Invitrogen, Taastrup, Denmark). Five μg total RNA was labelled using the SuperScript Choice System (Life Technologies), according to the manufacturer's instructions. Biotin-labelled cRNA was prepared using the BioArray High Yield RNA Transcript Labelling Kit (Enzo, Farmingdale, NY, USA), and 15 μg was loaded onto the probe array cartridge (Bovine Genome Array; Affymetrix, Santa Clara, CA, USA). The array contained 24128 probe sets which represented 15264 UniGene (annotation from May, 2006) to measure the global transcripts. The Bovine Genome Array annotation available from NetAffx™ Analysis Centre (Bovine.na29.annot.csv) was used. Additional and updated annotation was obtained from the Ensemble database, using the biomaRt package (version 2.0.0) in R [30]. In total 10401 probes on the array had a corresponding Ensemble ID, and 9369 probes were able to be assigned to the group Biological Processes (BP) of the Gene Ontology (GO) database http://www.geneontology.org/.

### Statistical analysis

The data was analyzed using R (version 2.10.0) http://www.r-project.org/. Normalization of the expression values for the udder samples was performed using the Robust Multi-array Average (RMA) algorithm as implemented in the Affy package (version 1.24.2). More detailed descriptions of the microarray experiment and data are available at the NCBI's Gene Expression Omnibus (GEO) database [31,32] through the accession number GSE24217.

Differential expression of each gene was assessed using linear modelling and empirical Bayes methods, which were implemented using the R package Limma (version 3.2.1) [33]. The linear models allowed for changes according to time points. Comparisons of the APR and chronic stage response were 24 h p.i. *vs*. 24 h control (T24 - C24) and 192 h p.i. *vs*. 192 h control (T192 - C192), respectively. Each transcript targeted by a probe was tested for its expression change using a modified Student's *t-*test. In the modified *t-*test, the residual standard deviations were moderated across the probe sets to ensure that there was a more stable inference for each transcript. The moderated standard deviations were considered as a compromise between the individual transcript standard deviations and the pooled transcripts standard deviation. Multiple testing was accounted for using the Bonferroni correction method. Probes were considered to be DE if the corrected *P*-value was below 0.05 and had a minimum log2-fold change of 1.0 for both the APR and chronic stage response.

Two-way clustering of the DE genes and samples (animals) was performed using the heatmap.2 option (gplots package version 2.7.4).

A hyper geometric gene set enrichment test (GOstats package version 2.12.0) was performed based on the clusters identified in the APR or chronic stage response comparison. Overrepresentation of gene sets defined by the group of BP in the GO database or the Kyoto Encyclopedia of Genes and Genomes database (KEGG) http://www.genome.jp/kegg/ was tested using Fisher's exact test. For this test, only the significant genes which were annotated with an Entrez gene ID were included. When a gene had a duplicate on the array, only a single gene ID was used. A gene-set was considered significant if *P *< 0.05.

The differentially expressed genes from our study at T = 24 h p.i. were compared to the *E. coli *expression studies (also at T = 24 h p.i.) previously reported in the literature [14,15]. The Affymetrix probe identifiers for the DE genes from the study by Mitterhuemer *et al*. [14] were taken from the GEO database (GSE15025), while the gene identifiers for and Rinaldi *et al*. [15] were taken from GSE15441). Expression levels were calculated at T = 24 h p.i. for all four regions in the udder mentioned in the study and a probe was included if it was differentially expressed in any of the regions. Since the arrays in Rinaldi *et al*. [15] were not Affymetrix, the probes were translated to Affymetrix probe IDs. Then, the annotated probes were matched with the list of annotated probes from our study and a Venn diagram was generated to show the overlap between the studies.

A hypergeometric gene set enrichment test was performed based on the KEGG pathways, as described earlier based on the DE genes that were common between our study, Mitterhuemer *et al*. [14], and Rinaldi *et al*. [15] (GOstats package version 2.12.0). A gene set was considered significant if *P *< 0.05.

## Authors' contributions

BB analyzed the microarray data and wrote the manuscript. CR carried out the animal challenges. KLI and CR collected the mammary biopsy samples. PS, CR and KLI designed the experimental plan. SME made the comparison between the gene lists of the different *E. coli *gene expression experiments available in the literature. BB, PS, CR, SME and KLI contributed to the interpretation of the results, discussion and refinement of the manuscript. The authors agreed on the contents of the paper.

## Supplementary Material

Additional file 1 **Table S1: Differentially expressed transcripts for the acute phase response to *E. coli *infection in the bovine udder**. Click here for file

Additional file 2 **Table S2: Significant GO identifiers detected based on the differentially expressed transcripts in cluster 1 for the acute phase response to *E. coli *infection**. A hypergeometric gene set enrichment test was performed based on cluster 1 of the contrast T24 *vs*. C24. Overrepresentation of gene sets defined by the GO database http://www.geneontology.org/ was tested using the Fisher's exact test. A gene set was considered significant if *P *< 0.05. Click here for file

Additional file 3 **Table S3: Significant GO identifiers detected based on the differentially expressed transcripts in cluster 2 for the acute phase response to *E. coli *infection**. A hypergeometric gene set enrichment test was performed based on cluster 2 of the contrast T24 *vs*. C24. A gene set was considered significant if *P *< 0.05. Click here for file

Additional file 4 **Figure S1: Venn diagram showing the overlap of differentially expressed genes between three studies on *E. coli *infection in the bovine udder at T = 24 h post-infection**. 1) "This study": the study described in this manuscript, 2) "Mitterhuemer": the study described by Mitterhuemer *et al*. [14], and 3) "Rinaldi": the study described by Rinaldi *et al*. [15]. Click here for file

Additional file 5 **Table S4: Overlap of differentially expressed genes between three studies of *E. coli *infection in the bovine udder**. Data from T = 24 h post-infection in three different studies: 1) "This study": the study described in this paper; 2) "Mitterhuemer": the study described by Mitterhuemer *et al*. [14]; and 3) "Rinaldi": the study described by Rinaldi *et al*. [15]. Click here for file
