# Supplementary material for: In depth analysis of genes and pathways of the mammary gland involved in the pathogenesis of bovine Escherichia coli-mastitis
Source: BMC Genomics. 2011 Feb 28;12:130. doi: 10.1186/1471-2164-12-130 (PMC3053262; doi:10.1186/1471-2164-12-130)
Supplement: Additional file 2 — Table S2: Significant GO identifiers detected based on the differentially expressed transcripts in cluster 1 for the acute phase response to E. coli infection. A hypergeometric gene set enrichment test was performed based on cluster 1 of the contrast T24 vs. C24. Overrepresentation of gene sets defined by the GO database http://www.geneontology.org/ was tested using the Fisher's exact test. A gene set was considered significant if P < 0.05. [file 1471-2164-12-130-S2.HTML]

Gene to GO BP Conditional test for over-representation

| GOBPID | Pvalue | OddsRatio | ExpCount | Count | Size | Term |
| GO:0006954 | 0.000 | 7.270 | 3 | 18 | 69 | inflammatory response |
| GO:0009605 | 0.000 | 3.574 | 8 | 25 | 172 | response to external stimulus |
| GO:0040011 | 0.000 | 3.765 | 6 | 20 | 127 | locomotion |
| GO:0006950 | 0.000 | 2.392 | 21 | 42 | 411 | response to stress |
| GO:0006935 | 0.000 | 8.951 | 1 | 9 | 29 | chemotaxis |
| GO:0007626 | 0.000 | 4.789 | 4 | 14 | 72 | locomotory behavior |
| GO:0050900 | 0.000 | 12.384 | 1 | 7 | 18 | leukocyte migration |
| GO:0006955 | 0.000 | 3.576 | 6 | 17 | 115 | immune response |
| GO:0031347 | 0.000 | 7.429 | 1 | 8 | 29 | regulation of defense response |
| GO:0032755 | 0.000 | 19.252 | 0 | 4 | 8 | positive regulation of interleukin-6 production |
| GO:0009617 | 0.000 | 7.921 | 1 | 6 | 21 | response to bacterium |
| GO:0050789 | 0.001 | 1.537 | 90 | 115 | 1805 | regulation of biological process |
| GO:0032101 | 0.001 | 4.715 | 2 | 8 | 41 | regulation of response to external stimulus |
| GO:0051240 | 0.001 | 4.177 | 3 | 9 | 51 | positive regulation of multicellular organismal process |
| GO:0032496 | 0.001 | 8.774 | 1 | 5 | 16 | response to lipopolysaccharide |
| GO:0060326 | 0.001 | 12.829 | 1 | 4 | 10 | cell chemotaxis |
| GO:0030593 | 0.001 | 28.773 | 0 | 3 | 5 | neutrophil chemotaxis |
| GO:0050901 | 0.003 | Inf | 0 | 2 | 2 | leukocyte tethering or rolling |
| GO:0042742 | 0.003 | 4.238 | 2 | 7 | 39 | defense response to bacterium |
| GO:0007264 | 0.003 | 2.250 | 9 | 18 | 176 | small GTPase mediated signal transduction |
| GO:0050766 | 0.004 | 14.381 | 0 | 3 | 7 | positive regulation of phagocytosis |
| GO:0050832 | 0.004 | 14.381 | 0 | 3 | 7 | defense response to fungus |
| GO:0048584 | 0.004 | 3.241 | 3 | 9 | 63 | positive regulation of response to stimulus |
| GO:0002253 | 0.004 | 4.638 | 2 | 6 | 31 | activation of immune response |
| GO:0030100 | 0.004 | 7.691 | 1 | 4 | 14 | regulation of endocytosis |
| GO:0006508 | 0.005 | 1.770 | 18 | 30 | 369 | proteolysis |
| GO:0051049 | 0.005 | 2.681 | 5 | 11 | 91 | regulation of transport |
| GO:0045454 | 0.007 | 3.995 | 2 | 6 | 35 | cell redox homeostasis |
| GO:0002224 | 0.007 | 38.218 | 0 | 2 | 3 | toll-like receptor signaling pathway |
| GO:0002758 | 0.007 | 38.218 | 0 | 2 | 3 | innate immune response-activating signal transduction |
| GO:0033089 | 0.007 | 38.218 | 0 | 2 | 3 | positive regulation of T cell differentiation in the thymus |
| GO:0042554 | 0.007 | 38.218 | 0 | 2 | 3 | superoxide anion generation |
| GO:0042590 | 0.007 | 38.218 | 0 | 2 | 3 | antigen processing and presentation of exogenous peptide antigen via MHC class I |
| GO:0046627 | 0.007 | 38.218 | 0 | 2 | 3 | negative regulation of insulin receptor signaling pathway |
| GO:0050896 | 0.008 | 2.248 | 7 | 14 | 156 | response to stimulus |
| GO:0006928 | 0.008 | 2.413 | 5 | 12 | 109 | cell motion |
| GO:0001817 | 0.009 | 3.763 | 2 | 6 | 37 | regulation of cytokine production |
| GO:0019884 | 0.011 | 8.212 | 1 | 3 | 10 | antigen processing and presentation of exogenous antigen |
| GO:0051048 | 0.011 | 8.212 | 1 | 3 | 10 | negative regulation of secretion |
| GO:0009056 | 0.014 | 1.523 | 29 | 41 | 582 | catabolic process |
| GO:0002861 | 0.014 | 19.105 | 0 | 2 | 4 | regulation of inflammatory response to antigenic stimulus |
| GO:0042116 | 0.014 | 19.105 | 0 | 2 | 4 | macrophage activation |
| GO:0042891 | 0.014 | 19.105 | 0 | 2 | 4 | antibiotic transport |
| GO:0043154 | 0.014 | 19.105 | 0 | 2 | 4 | negative regulation of caspase activity |
| GO:0045986 | 0.014 | 19.105 | 0 | 2 | 4 | negative regulation of smooth muscle contraction |
| GO:0050728 | 0.015 | 7.184 | 1 | 3 | 11 | negative regulation of inflammatory response |
| GO:0042592 | 0.019 | 1.818 | 11 | 18 | 212 | homeostatic process |
| GO:0007165 | 0.019 | 1.430 | 40 | 52 | 805 | signal transduction |
| GO:0002429 | 0.020 | 6.385 | 1 | 3 | 12 | immune response-activating cell surface receptor signaling pathway |
| GO:0006826 | 0.020 | 6.385 | 1 | 3 | 12 | iron ion transport |
| GO:0006953 | 0.020 | 6.385 | 1 | 3 | 12 | acute-phase response |
| GO:0048002 | 0.020 | 6.385 | 1 | 3 | 12 | antigen processing and presentation of peptide antigen |
| GO:0050871 | 0.020 | 6.385 | 1 | 3 | 12 | positive regulation of B cell activation |
| GO:0045321 | 0.021 | 2.741 | 3 | 7 | 57 | leukocyte activation |
| GO:0006911 | 0.023 | 12.734 | 0 | 2 | 5 | phagocytosis, engulfment |
| GO:0034097 | 0.023 | 12.734 | 0 | 2 | 5 | response to cytokine stimulus |
| GO:0045055 | 0.023 | 12.734 | 0 | 2 | 5 | regulated secretory pathway |
| GO:0051224 | 0.023 | 12.734 | 0 | 2 | 5 | negative regulation of protein transport |
| GO:0016337 | 0.023 | 2.964 | 2 | 6 | 45 | cell-cell adhesion |
| GO:0042981 | 0.025 | 1.907 | 8 | 14 | 157 | regulation of apoptosis |
| GO:0043285 | 0.025 | 1.538 | 21 | 30 | 416 | biopolymer catabolic process |
| GO:0007565 | 0.026 | 4.040 | 1 | 4 | 23 | female pregnancy |
| GO:0010941 | 0.027 | 1.880 | 8 | 14 | 159 | regulation of cell death |
| GO:0010646 | 0.028 | 1.725 | 11 | 18 | 222 | regulation of cell communication |
| GO:0002252 | 0.032 | 2.500 | 3 | 7 | 61 | immune effector process |
| GO:0008283 | 0.032 | 1.699 | 11 | 18 | 225 | cell proliferation |
| GO:0002696 | 0.032 | 3.100 | 2 | 5 | 36 | positive regulation of leukocyte activation |
| GO:0009620 | 0.032 | 9.657 | 0 | 2 | 6 | response to fungus |
| GO:0001829 | 0.033 | 9.548 | 0 | 2 | 6 | trophectodermal cell differentiation |
| GO:0006020 | 0.033 | 9.548 | 0 | 2 | 6 | inositol metabolic process |
| GO:0018149 | 0.033 | 9.548 | 0 | 2 | 6 | peptide cross-linking |
| GO:0031649 | 0.033 | 9.548 | 0 | 2 | 6 | heat generation |
| GO:0045089 | 0.033 | 9.548 | 0 | 2 | 6 | positive regulation of innate immune response |
| GO:0045351 | 0.033 | 9.548 | 0 | 2 | 6 | type I interferon biosynthetic process |
| GO:0050707 | 0.033 | 9.548 | 0 | 2 | 6 | regulation of cytokine secretion |
| GO:0070201 | 0.034 | 3.654 | 1 | 4 | 25 | regulation of establishment of protein localization |
| GO:0009968 | 0.035 | 3.027 | 2 | 5 | 37 | negative regulation of signal transduction |
| GO:0002822 | 0.036 | 4.785 | 1 | 3 | 15 | regulation of adaptive immune response based on somatic recombination of immune receptors built from immunoglobulin superfamily domains |
| GO:0006937 | 0.036 | 4.785 | 1 | 3 | 15 | regulation of muscle contraction |
| GO:0030036 | 0.037 | 2.409 | 3 | 7 | 63 | actin cytoskeleton organization |
| GO:0051605 | 0.039 | 3.487 | 1 | 4 | 26 | protein maturation by peptide bond cleavage |
| GO:0007155 | 0.039 | 1.878 | 7 | 12 | 138 | cell adhesion |
| GO:0050776 | 0.043 | 2.529 | 3 | 6 | 52 | regulation of immune response |
| GO:0052548 | 0.043 | 4.416 | 1 | 3 | 16 | regulation of endopeptidase activity |
| GO:0010033 | 0.043 | 2.061 | 5 | 9 | 94 | response to organic substance |
| GO:0051707 | 0.044 | 4.340 | 1 | 3 | 17 | response to other organism |
| GO:0032760 | 0.044 | 7.637 | 0 | 2 | 7 | positive regulation of tumor necrosis factor production |
| GO:0050729 | 0.044 | 7.637 | 0 | 2 | 7 | positive regulation of inflammatory response |
| GO:0016265 | 0.045 | 1.582 | 13 | 20 | 267 | death |
| GO:0010740 | 0.049 | 3.195 | 1 | 4 | 28 | positive regulation of protein kinase cascade |
| GO:0050867 | 0.049 | Inf | 0 | 1 | 1 | positive regulation of cell activation |
| GO:0002768 | 0.050 | Inf | 0 | 1 | 1 | immune response-regulating cell surface receptor signaling pathway |
