# Supplementary material for: In depth analysis of genes and pathways of the mammary gland involved in the pathogenesis of bovine Escherichia coli-mastitis
Source: BMC Genomics. 2011 Feb 28;12:130. doi: 10.1186/1471-2164-12-130 (PMC3053262; doi:10.1186/1471-2164-12-130)
Supplement: Additional file 3 — Table S3: Significant GO identifiers detected based on the differentially expressed transcripts in cluster 2 for the acute phase response to E. coli infection. A hypergeometric gene set enrichment test was performed based on cluster 2 of the contrast T24 vs. C24. A gene set was considered significant if P < 0.05. [file 1471-2164-12-130-S3.HTML]

Gene to GO BP Conditional test for over-representation

| GOBPID | Pvalue | OddsRatio | ExpCount | Count | Size | Term |
| GO:0019752 | 0.001 | 4.324 | 2 | 9 | 232 | carboxylic acid metabolic process |
| GO:0042180 | 0.001 | 4.205 | 3 | 9 | 238 | cellular ketone metabolic process |
| GO:0055114 | 0.002 | 3.300 | 4 | 10 | 335 | oxidation reduction |
| GO:0042326 | 0.006 | 21.473 | 0 | 2 | 11 | negative regulation of phosphorylation |
| GO:0006082 | 0.006 | 4.018 | 2 | 6 | 166 | organic acid metabolic process |
| GO:0010563 | 0.007 | 19.322 | 0 | 2 | 12 | negative regulation of phosphorus metabolic process |
| GO:0006633 | 0.007 | 8.403 | 0 | 3 | 38 | fatty acid biosynthetic process |
| GO:0006629 | 0.008 | 3.109 | 3 | 8 | 275 | lipid metabolic process |
| GO:0006469 | 0.008 | 17.561 | 0 | 2 | 13 | negative regulation of protein kinase activity |
| GO:0006639 | 0.008 | 17.561 | 0 | 2 | 13 | acylglycerol metabolic process |
| GO:0051348 | 0.008 | 17.561 | 0 | 2 | 13 | negative regulation of transferase activity |
| GO:0006631 | 0.010 | 7.386 | 0 | 3 | 45 | fatty acid metabolic process |
| GO:0005997 | 0.011 | Inf | 0 | 1 | 1 | xylulose metabolic process |
| GO:0006482 | 0.011 | Inf | 0 | 1 | 1 | protein amino acid demethylation |
| GO:0010744 | 0.011 | Inf | 0 | 1 | 1 | positive regulation of foam cell differentiation |
| GO:0015820 | 0.011 | Inf | 0 | 1 | 1 | leucine transport |
| GO:0015823 | 0.011 | Inf | 0 | 1 | 1 | phenylalanine transport |
| GO:0015827 | 0.011 | Inf | 0 | 1 | 1 | tryptophan transport |
| GO:0015993 | 0.011 | Inf | 0 | 1 | 1 | molecular hydrogen transport |
| GO:0032429 | 0.011 | Inf | 0 | 1 | 1 | regulation of phospholipase A2 activity |
| GO:0032431 | 0.011 | Inf | 0 | 1 | 1 | activation of phospholipase A2 activity |
| GO:0034367 | 0.011 | Inf | 0 | 1 | 1 | macromolecular complex remodeling |
| GO:0034369 | 0.011 | Inf | 0 | 1 | 1 | plasma lipoprotein particle remodeling |
| GO:0034372 | 0.011 | Inf | 0 | 1 | 1 | very-low-density lipoprotein particle remodeling |
| GO:0048662 | 0.011 | Inf | 0 | 1 | 1 | negative regulation of smooth muscle cell proliferation |
| GO:0018904 | 0.011 | 14.854 | 0 | 2 | 15 | organic ether metabolic process |
| GO:0000188 | 0.021 | 94.923 | 0 | 1 | 2 | inactivation of MAPK activity |
| GO:0006651 | 0.021 | 94.923 | 0 | 1 | 2 | diacylglycerol biosynthetic process |
| GO:0046325 | 0.021 | 94.923 | 0 | 1 | 2 | negative regulation of glucose import |
| GO:0048511 | 0.028 | 8.379 | 0 | 2 | 25 | rhythmic process |
| GO:0006422 | 0.032 | 47.452 | 0 | 1 | 3 | aspartyl-tRNA aminoacylation |
| GO:0042732 | 0.032 | 47.452 | 0 | 1 | 3 | D-xylose metabolic process |
| GO:0046627 | 0.032 | 47.452 | 0 | 1 | 3 | negative regulation of insulin receptor signaling pathway |
| GO:0016053 | 0.032 | 4.642 | 1 | 3 | 66 | organic acid biosynthetic process |
| GO:0019748 | 0.040 | 6.875 | 0 | 2 | 30 | secondary metabolic process |
| GO:0001938 | 0.042 | 31.628 | 0 | 1 | 4 | positive regulation of endothelial cell proliferation |
| GO:0009303 | 0.042 | 31.628 | 0 | 1 | 4 | rRNA transcription |
| GO:0019433 | 0.042 | 31.628 | 0 | 1 | 4 | triglyceride catabolic process |
| GO:0044269 | 0.042 | 31.628 | 0 | 1 | 4 | glycerol ether catabolic process |
| GO:0046460 | 0.042 | 31.628 | 0 | 1 | 4 | neutral lipid biosynthetic process |
| GO:0046461 | 0.042 | 31.628 | 0 | 1 | 4 | neutral lipid catabolic process |
| GO:0046503 | 0.042 | 31.628 | 0 | 1 | 4 | glycerolipid catabolic process |
| GO:0046942 | 0.042 | 6.637 | 0 | 2 | 31 | carboxylic acid transport |
