# Supplementary figures and images for: In depth analysis of genes and pathways of the mammary gland involved in the pathogenesis of bovine Escherichia coli-mastitis
Source: BMC Genomics. 2011 Feb 28;12:130. doi: 10.1186/1471-2164-12-130 (PMC3053262; doi:10.1186/1471-2164-12-130)

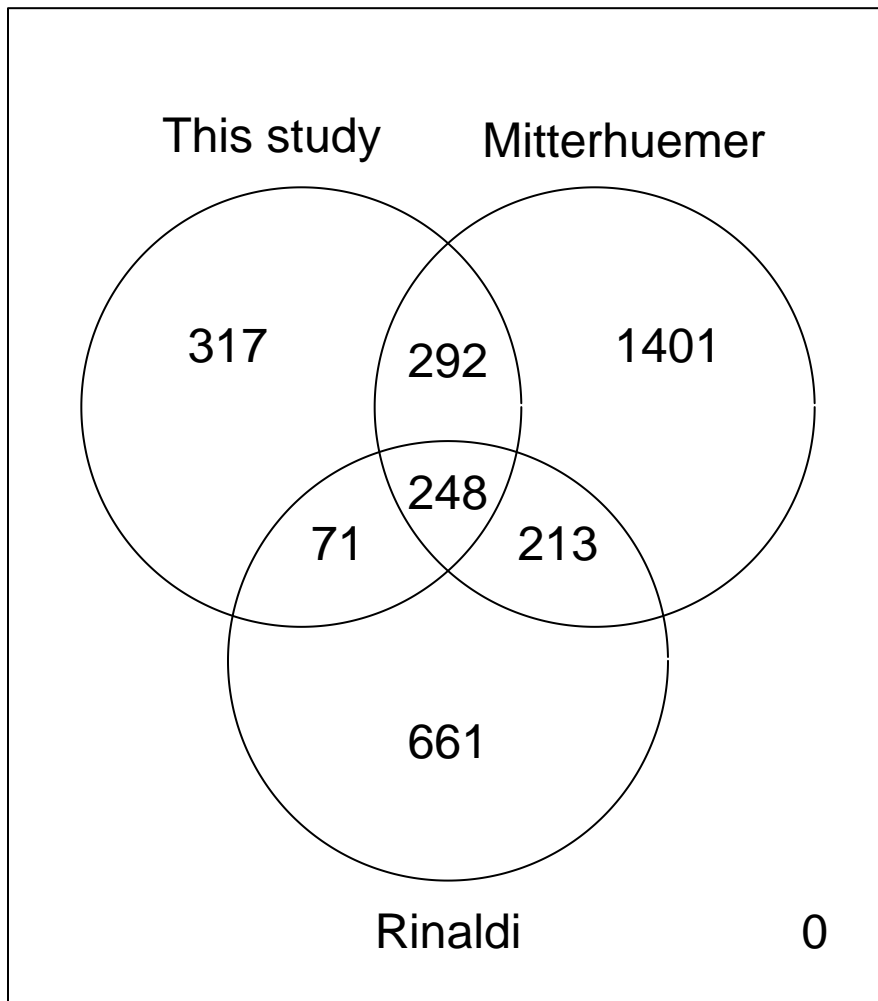

Supplement: Additional file 4 — Figure S1: Venn diagram showing the overlap of differentially expressed genes between three studies on E. coli infection in the bovine udder at T = 24 h post-infection. 1) "This study": the study described in this manuscript, 2) "Mitterhuemer": the study described by Mitterhuemer et al. [14], and 3) "Rinaldi": the study described by Rinaldi et al. [15]. [file 1471-2164-12-130-S4.PDF]
